# Artificial Intelligence in Various Medical Fields With Emphasis on Radiology: Statistical Evaluation of the Literature

**DOI:** 10.7759/cureus.10961

**Published:** 2020-10-15

**Authors:** Emre Pakdemirli, Urszula Wegner

**Affiliations:** 1 Radiology, St. Albans City Hospital, West Hertfordshire Hospitals NHS Trust, St. Albans, GBR; 2 Radiology, King George Hospital, Barking, Havering and Redbridge University Hospitals, London, GBR

**Keywords:** machine learning, artificial intelligence, medicine, radiology, bibliometric analysis

## Abstract

Background

Artificial intelligence (AI) has significantly impacted numerous medical specialties with high emphasis on radiology. Associated novel diagnostic methods have become a rapidly emerging hot topic, and it is essential to provide insights into quantitative analysis of the growing literature.

Purpose

The purpose of this study is to highlight future academic trends, identify potential research gaps, and analyze scientific landscape of AI in the field of medicine.

The main aim is to explore comprehensive dataset over a 46-year period in terms of publication type, publication citation, country of origin, institution, and medical specialty.

Material and Methods

The Web of Science database was searched from 1975 to 2020, and publications on AI were explored. Both original research reports and review articles were included in comprehensive bibliometric analysis. Descriptive statistics were calculated, and numerous variables were applied, namely year of publication, institution, type of publication, specialty area, country of origin, and citation numbers, and the Kruskal-Wallis analysis of variance was used.

Results

A total of 117,974 relevant citations were retrieved, of which 83,979 original research and review articles were retained for analysis. Not surprisingly, the largest proportion of citations were from the United States (23%, n = 19,180) followed by China, Spain, England, and Germany. The number of citations was relatively consistent during the 1970s and emerging gradually during the 1980s. However, ongoing scientific trend positively evolved, and the numbers started to grow significantly in the 1990s and demonstrated continuous increasing wave since then. The most frequently represented key medical specialties were oncology, radiology, neuroradiology, and ophthalmology. Overall, no major statistical difference was found between these four domains (p = 0.753).

Conclusions

In summary, research on AI-powered technologies in the medical domain was at early stage in the 1970s. However, associated deep learning algorithms significantly attracted and revolutionized the scientific community with subsequent evolution of research and exponential growth of multidisciplinary publications since that time. Work in this field has impacted radiology as an area of predominant interest and has been led by institutions in the United States, Spain, France, China, and England. The bibliometric study reported herein can provide a broad overview and valuable guidance to help medical researchers gain insights into key points and trace the global trends regarding the status of AI research in medicine, particularly in radiology and other relevant multispecialty areas.

## Introduction

Artificial intelligence (AI) powered technologies are used in almost all sectors and fields of modern life. A ground-breaking 1971 study by Nordyke [1] is generally considered to have inaugurated the field of AI in medicine [2], and the implementation of AI in general medical applications and in specialties, such as radiology, oncology, and pathology, has grown in recent years. AI-enhanced automated devices are ubiquitous in the health sector in industrialized and developing countries, where the field is growing in response to healthcare worker shortages. AI-enhanced algorithms are particularly valuable in such settings, especially for diagnostics. For example, while simple chest X-ray examinations are most accurately interpreted by a thorough examination from a radiologist, the rate of clinically significant errors by radiologists is in the range of 2-20% [3]. Interpretation of chest X-rays by non-radiologists would likely be even more error-prone, leading to negative impacts on patient care and prognosis.

AI in medicine has become an increasingly important topic and one of the leading directions in medicine with particular emphasis on the field of radiology. Despite concerns and skepticism of some physicians, the use of AI in medicine is growing across the health sector, and its benefits have been thoroughly described by several medical societies globally. Given these positive trends, it is likely that healthcare workers will be using AI to an increasing extent in the near future [4]. Reports on AI in radiology have been led in recent years by the Radiological Society of North America (RSNA). The reliability and acceptance of AI in radiology are growing, and research publication in the field has been particularly accelerated since 2015 [5]. Radiology accounts for nearly 90% of “big data” usage in medicine. There has been copious discussion in recent years regarding the position of AI in medicine, with particular focus on the management of big data, evaluation of algorithms, and medico-legal issues. The U.S. Food and Drug Administration (FDA) has already endorsed several AI algorithms for the benefit of patients and physicians, and several other organizations have followed the FDA’s initiatives. If such algorithms work properly for narrow tasks, they are unlikely to be outperformed by human labor.

Researchers have written extensively on the benefits of AI applications in medicine, emphasizing the potential of this technology to improve diagnostic accuracy, therapeutic efficacy, and the overall clinical treatment process [6]. AI applications have assisted doctors and other medical professionals in numerous domains including health information systems, geocoding of health data, epidemic and syndromic surveillance, predictive models, decision support, and medical imaging [7-11]. AI systems are capable of providing health professionals with continuous real-time updates on medical information from various sources, including journals, textbooks, clinical practices, and patients, enabling improved patient care and supporting appropriate inferences for health risk assessment and prediction of health outcomes [9,10].

AI is rapidly transforming the medical landscape, and research in the field has expanded dramatically in recent decades, thus presenting the need for a comprehensive review describing research patterns and trends over time.

The purpose of this study is to analyze and describe the scientific literature on AI in medicine over a 46-year period in terms of publication type, publication citation, country of origin, institution, and medical specialty.

## Materials and methods

Search strategy criteria were used to construct a bibliometric research framework. We summarized the two-step search strategy (Table 1). The Web of Science (WoS) database (SCI Expanded and SSCI) were extensively searched regarding the timeframe of 1975-2020. Numerous scientific publications on the subject of AI were retrieved for the study purposes. Original research articles and reviews were retained for analysis. Books and book chapters, conference proceedings, early access and retracted publications, and citation reports were excluded from the study. Data were collected on publication year, institution, country of origin, publication type, and medical specialty area using the “analyse result and create citation report” function in WoS. Data were analyzed using SPSS Version 24.0 (IBM Corp., Armonk, NY, USA). Descriptive statistics were calculated and rank-based nonparametric Kruskal-Wallis tests were used. A p-value of <0.05 was considered statistically significant.

**Table 1:** WoS search strategy and results WoS, Web of Science

WoS Search Keywords
Step 1
Topic: (“Artificial Intelligence”)
Time frame: 1975-2020
Indexes: SCI-EXPANDED, SSCI, AHCI, CPCI-S, CPCI-SSH, BKCI-S, BKCI-SSH, ESCI.
Document types: Article, review
Results: 86,908 publications (83,979 Article, 2929 Review)
Step 2
In addition to the above information:
Subtopic: (“Radiology Nuclear Medicine Medical Imaging”)
Results: 937 publications (1.08%)
Subtopic: (“Oncology”)
Results: 316 publications (0.37%)
Subtopic: (“Neuroimaging”)
Results: 151 publications (0.17%)
Subtopic: (“Ophthalmology”)
Results: 139 publications (0.16%)

**Figure 1:**
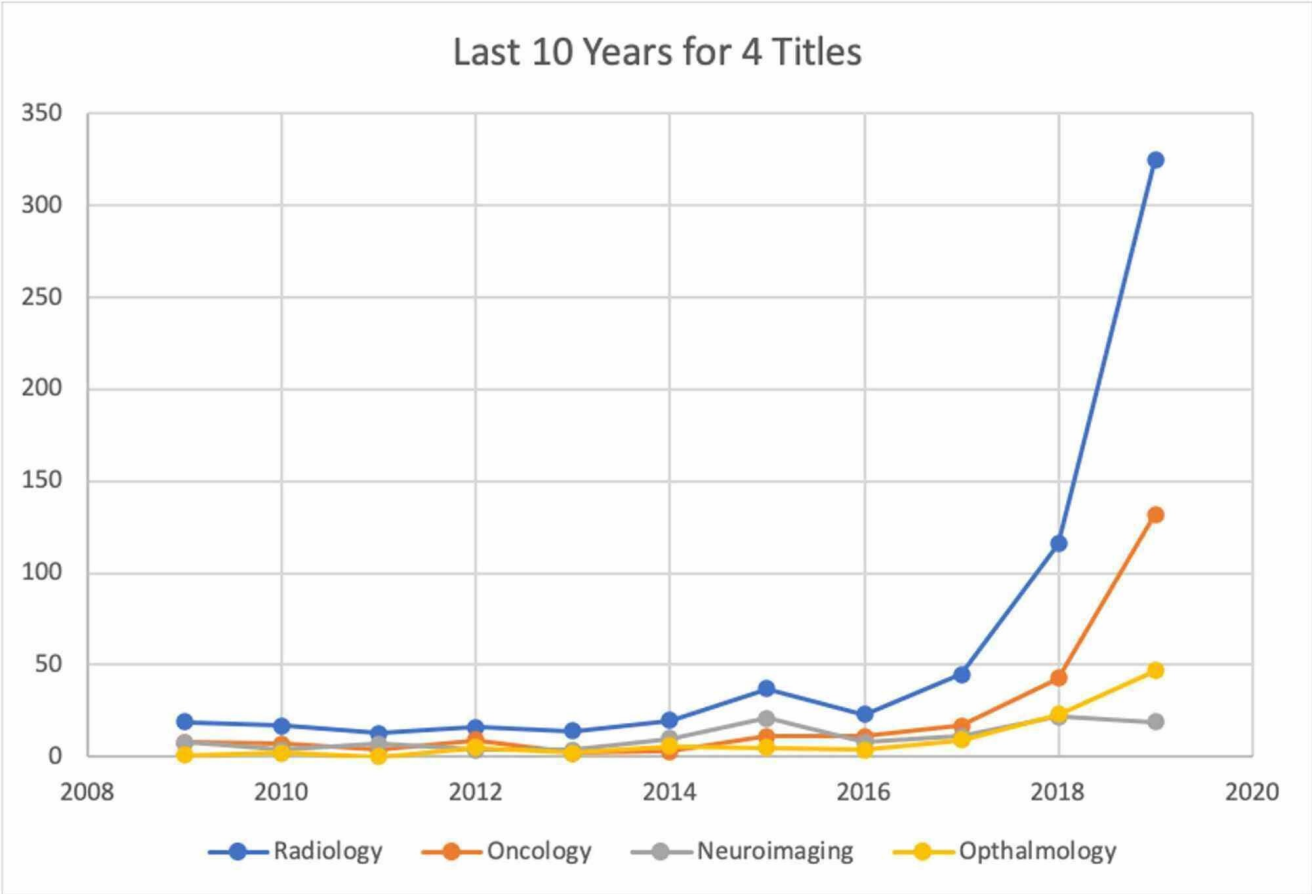
Publication figures for four medical specialties, 2009–2019

WoS includes 250 categories under the heading of AI. We investigated relevant multispecialty medical sciences (e.g., radiology, ophthalmology, neuroimaging, oncology, pathology, otorhinolaryngology, dermatology). The distribution of research publications across medical fields within the 250 categories was calculated, and further descriptive statistics were calculated for radiology, oncology, ophthalmology, and neuroimaging (Figure 1).

## Results

**Figure 2:**
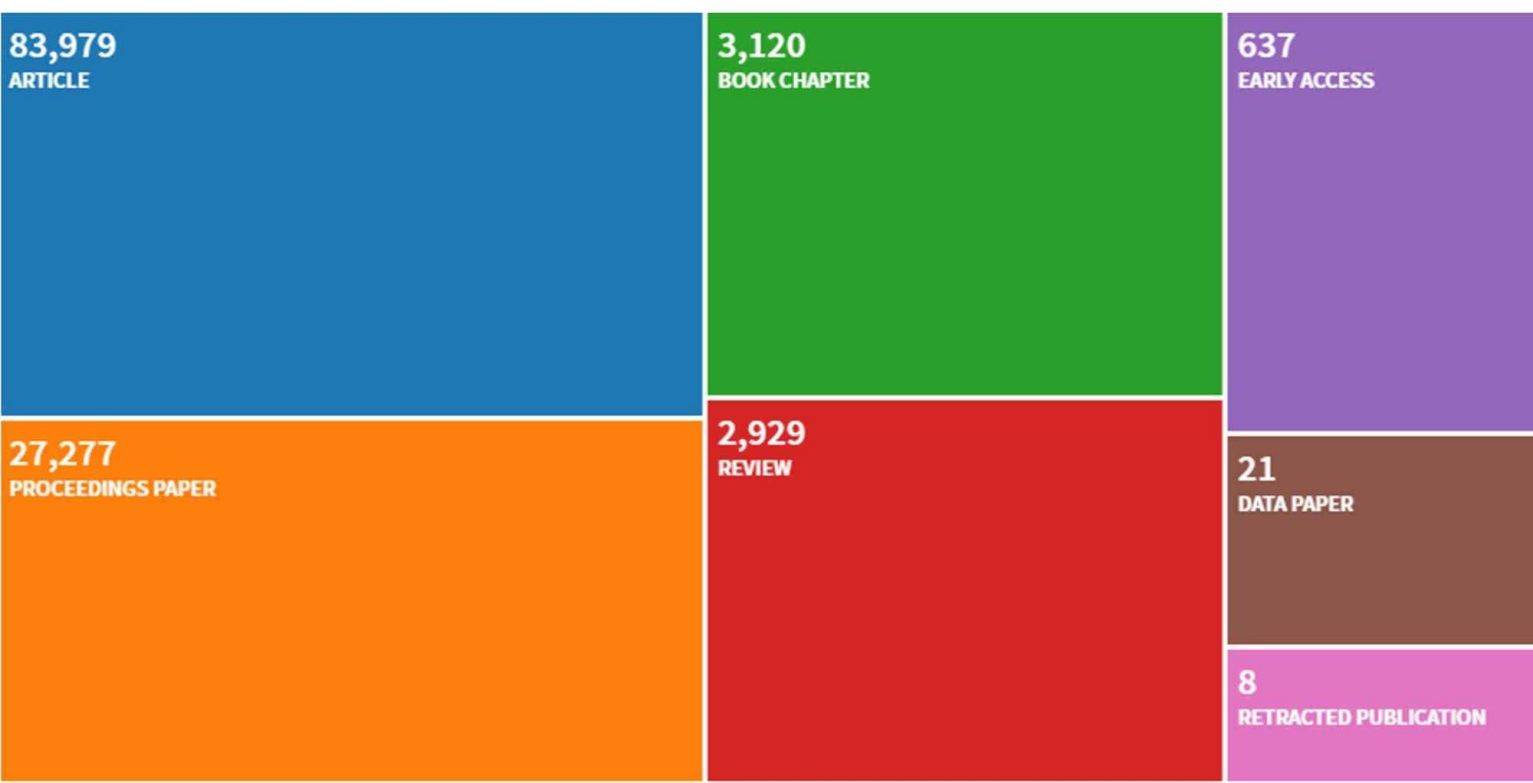
Document types

A total of 117,974 documents on AI were retrieved across all fields. As shown in Figure 2, 71.18% of these documents were original research articles (n = 83,979), 23.12% were proceedings papers (n = 27,277), 2.64% were book chapters (n = 3,120), 2.48% were reviews (n = 2,929), and 0.56% were other types of documents (n = 669). Research articles and reviews (n = 86,908) were retained for analyses (Figure 2).

**Figure 3:**
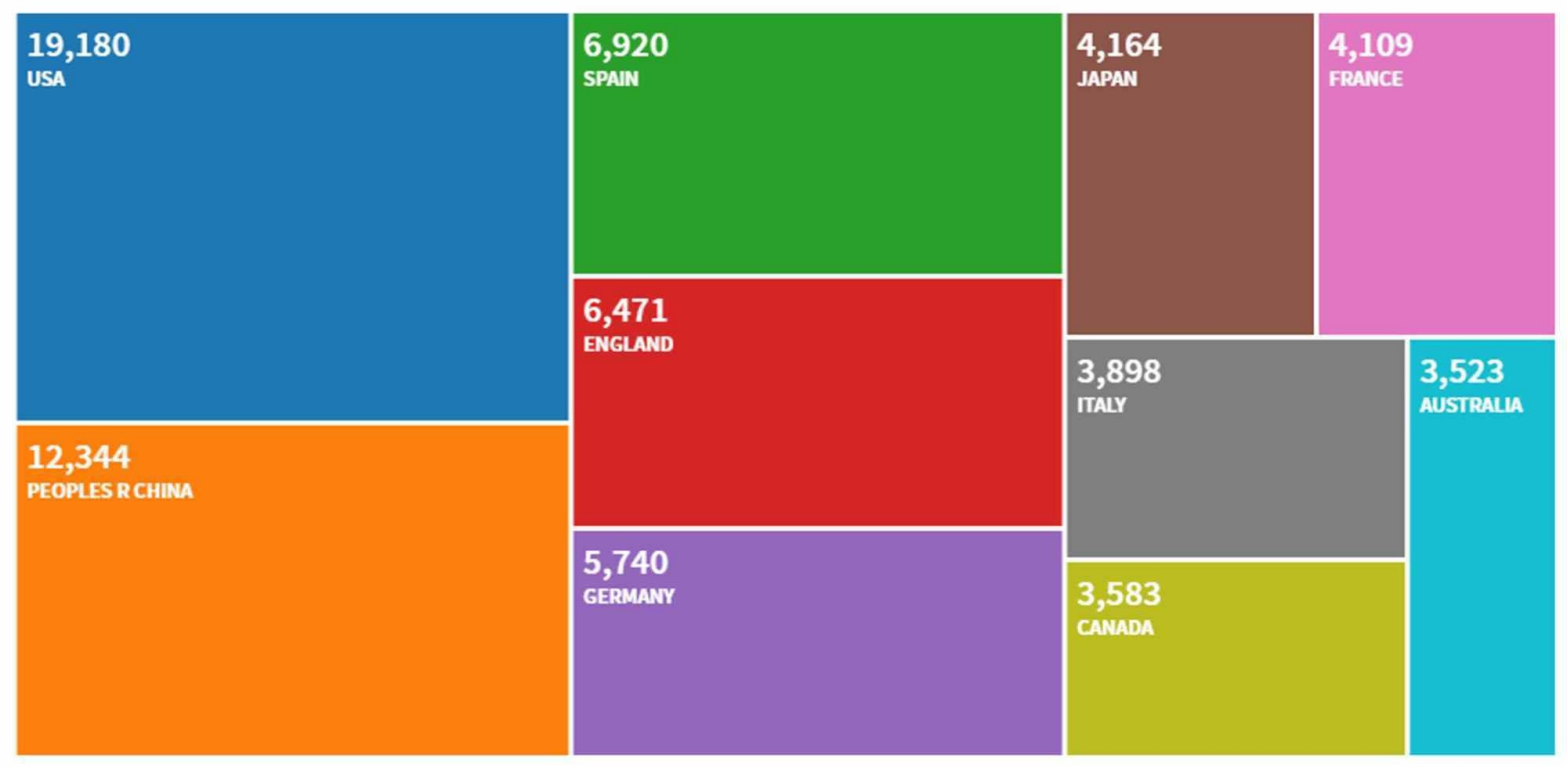
Article distribution by country

Not surprisingly, the largest proportion of citations were from the United States (42.32%, n = 19,180). However, numerous citations were from other countries, accounting for more than 5% of citations from China (14.2%, n = 12,344), Spain (7.96%, n = 6,920), England (7.44%, n = 6,471), and Germany (6.6%, n = 5,740) (Figure 3).

**Figure 4:**
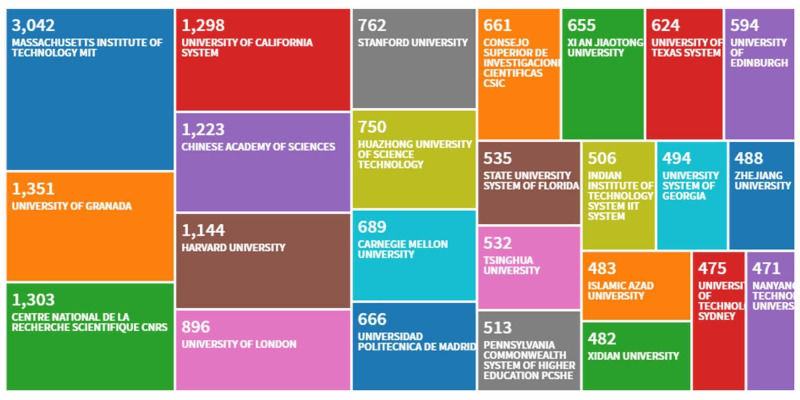
Article distribution by institution

Figure 4 shows the distribution of citations by institution with regard to the top 25 influential institutions. The top 10 institutions were located in the United States, Spain, France, China, and England. Interestingly, the largest number of citations came from the Massachusetts Institute of Technology (MIT) (n = 3,042 studies). Other institutions with more than 1,000 citations included the University of Granada in Spain (n = 1,351), the Centre national de la recherche scientifique in France (n = 1,303), the University of California System (n = 1,298), and the Chinese Academy of Sciences (n = 1,223).

The number of citations by publication year is shown in Table 2. During the four years from 1974 to 1977, there was no increase in the number of studies on AI in medicine. The number of citations increased gradually during the 1980s, rose more dramatically during the 1990s, and has continued to grow since that time.

**Table 2:** Article distribution by year of publication (N = 86,908)

Year of publication	Records	Percent
2020	1312	1.51
2019	10673	12.28
2018	6202	7.14
2017	4178	4.81
2016	3544	4.08
2015	3149	3.62
2014	2511	2.89
2013	2330	2.68
2012	2076	2.39
2011	1972	2.27
2010	1830	2.11
2009	1935	2.23
2008	1773	2.04
2007	1562	1.80
2006	5877	6.76
2005	7138	8.21
2004	3815	4.39
2003	4321	4.97
2002	2555	2.94
2001	1835	2.11
2000	2253	2.59
1999	2064	2.38
1998	1768	2.03
1997	1683	1.94
1996	1152	1.33
1995	1384	1.59
1994	849	0.98
1993	825	0.95
1992	1185	1.36
1991	928	1.07
1990	359	0.41
1989	260	0.30
1988	288	0.33
1987	281	0.32
1986	254	0.29
1985	254	0.29
1984	148	0.17
1983	115	0.13
1982	87	0.10
1981	88	0.10
1980	73	0.08
1979	15	0.02
1978	3	<0.01
1977	1	<0.01
1976	1	<0.01
1975	1	<0.01
1974	1	<0.01

The trends from 2009 to 2019 in the number of citations in the four domains of interest are shown in Figure 1. Citations in radiology increased dramatically over the last two years, whereas citations in the other three fields rose more moderately. There was no statistically significant difference between the four selected specialties in medicine in terms of publications over the last 10 years based on the Kruskal-Wallis test (Χ2 = 1.198; df = 3; p = 0.753) (Table 3).

**Table 3:** Kruskal-Wallis statistical calculation for four medical specialties *There is no statistical significance between four selected specialties in terms of difference in ratio in the last 10 years publications according to the Kruskal-Wallis H test (Χ2 =1.198; df = 3; p = 0.753).

Percentage	Group	n	Mean rank	Χ^2^	df	p-Value
% change in terms of articles and reviews	Radiology	10	20.35	1.198	3	0.753*
	Oncology	10	22.2			
	Neuroimaging	10	17.25			
	Ophthalmology	10	22.2			

## Discussion

We conducted a bibliometric analysis of the scientific literature related to AI-powered technologies in medicine over a 46-year period with the main aim of exploring associated ongoing research trends in radiology and other medical specialties. Our findings indicate that the number of papers published in medical AI has been increasing substantially during the study period, with a particularly sharp increase seen over the last several years. We identified a total of 11 articles for both 2016 and 2017, as well as 53 records in 2018. Similar trends appear to be taking place in 2020, though we were able to include data only from the beginning of the year. Our findings show that scholarly publication pertaining to AI is growing in the field of radiology and medicine more generally. Since AI is clearly an important hot topic that has garnered wide interest across numerous fields, related research is particularly extensive and rapidly growing in the areas of oncology, radiology, ophthalmology, and neuroimaging.

We were able to identify two English-language bibliometric studies [10,11] conducted to date on AI in medicine. Kulakli and Osmanaj [10] conducted a review of research studies on big data in relation to AI published in peer-reviewed journals and indexed in WoS for the period of 2008-2019. Their study covers a wider range than ours and included non-medical fields such as engineering, telecommunications, computer science, and law. They also examined citation results for the included publications, which we did not investigate. Our review was more narrowly focused on the medical field but covered a much wider timeframe and was not limited to literature specifically on big data. While we did not investigate particular journals or authors, we did analyse the search results to determine the most productive institutions. Findings from the review by Kulakli and Osmanaj show some similar patterns to ours, including a preponderance of studies from the United States. In addition, we found a dramatic jump in the number of relevant publications between 2018 and 2019, which was also observed in Kulakli and Osmanaj’s review. The authors concluded that research on big data in relation to AI is expanding, a pattern also evident in our findings.

Tran et al. [11] conducted a comprehensive bibliometric study of AI in medicine in which they recommended the development of global and national protocols and regulations for the justification and adaptation of medical AI products. Their analysis focused on articles published in peer-reviewed journals from 1971 to 2018 based on data from WoS and Scopus. Their study does not include grey literature, conference proceedings or books/book chapters. As with our study, articles not in English were excluded. Their final analytic sample of 27,451 articles was substantially smaller than ours. However, they performed analyses based on authors, citations, key words, and diseases of interest. Similar to the results from our study, the five most frequently featured countries were in North America (the United States and Canada), Europe (Italy and Germany), and China.

Our study is subject to a few limitations. While we included both original research and review papers, the analyses were limited to English-language publications indexed in the WoS database, and we did not include other types of publications such as books, book chapters, and conference proceedings. Conducted bibliometric analysis depicts trends in research in the field of AI in medicine and provides a detailed view of publications in relevant specialty areas. Future studies will be needed to track new developments in this important and growing field.

## Conclusions

Academic literature on AI in medicine had little room for optimism through the late 1970s. However, AI-driven software soon influenced and inspired qualified staff across the globe with subsequent increase of numerous associated publications in the 1990s. Importantly, this positive research trend demonstrates continuous dramatic growth pattern over the recent years.

Since AI may have the potential to empower healthcare with novel diagnostic tools, influence decision-making or even outperform clinicians, it is crucial to shed light on the bibliometrics of the growing literature and address any potential or explored scientific gaps.

To the best of our knowledge, the presented review of bibliographic data represents one of the most comprehensive analysis in terms of data coverage and timeframe with regard to the evolution of the academic quality related to the field of enquiry. Notably, the study illustrates substantial progress in the academic realm with regard to AI-driven algorithms in the fields of oncology, radiology, neuroradiology, and ophthalmology.

In summary, our statistical evaluation provides a better understanding of the fundamentals of ongoing research and directions for the future and shares knowledge on current global trends with the scientific community.
